# Defining an integrative multidimensional framework for patient complaint management systems

**DOI:** 10.3389/frhs.2026.1767605

**Published:** 2026-03-30

**Authors:** Aydin Teymourifar

**Affiliations:** Department of Industrial Engineering, Istanbul Sabahattin Zaim University, Istanbul, Türkiye

**Keywords:** complaint management, hospital systems, organisational learning, patient complaints, proactive risk detection

## Abstract

Patient complaints represent a significant yet sometimes underutilized source of organisational learning in hospitals. While they are sometimes processed as administrative tasks aimed at resolution, emerging research demonstrates their value as indicators of system vulnerability and early warning signals for patient safety. This mini-review contributes to the literature by offering a new integrative framework that synthesizes eight previously fragmented domains of complaint management: System Consistency in Learning from Complaints, standard classification of complaints, leadership commitment for organisational learning, effective individual and interpersonal behaviour for organisational learning, organisational change driven by organisational learning, data analytics for organisational learning, proactive risk detection, ethical and legal considerations. Using an integrative, framework-informed methodology, the paper combines inductive identification of conceptual patterns with deductive synthesis across theoretical and policy literatures. The review's key contribution lies in reconceptualizing patient complaints as a system embedded within hospital operations, rather than an administrative function. By mapping connections across the eight domains, the framework reveals how complaints serve as multidimensional organisational assets that can expose systemic weaknesses, cultural blind spots, and latent safety risks. The review also identifies significant theoretical and measurement gaps, including the lack of validated multidimensional instruments to assess complaint management holistically. It further proposes a new research direction by outlining how performance-based contracting could align incentives among patients, clinicians, and managers to strengthen responsiveness and learning, with implications for health system governance and public health oversight.

## 1 Introduction

Hospitals increasingly emphasize patient-centered care, yet patients’ voices are sometimes marginalized in organisational decision-making (1–4, 87, 88). Patient complaints, rich accounts of dissatisfaction, unmet expectations, and emerging risks continue to be processed as administrative tasks aimed at closure rather than as signals capable of revealing deeper systemic vulnerabilities (5–10). While complaints can expose communication failures and safety concerns that conventional indicators overlook (11–17), many institutions neutralize them through bureaucratic routines that favour resolution over reflection and compliance over insight. The result is a persistent disconnect between the experiential knowledge embedded in complaints and the organisational learning they could enable (18–22).

Research on patient complaints, although growing, remains fragmented across disciplinary silos. Systematic reviews have demonstrated a substantial and expanding evidence base. Still, this work is characterised by heterogeneous aims, methods, and settings, with scholars calling for standardised taxonomies to support comparison and synthesis (5, 6, 17, 23). This fragmentation is also visible in how scholars conceptualise complaints: some treat them primarily as indicators of patient experience or quality metrics within improvement systems (24–26), while others position complaints as part of medico-legal processes that may signal potentially compensable incidents or claims (27–29). Complaints have further been framed as performance indicators within broader patient safety and surveillance architectures, listed alongside mortality, infection rates and other system-level metrics (30, 91), and as safety-critical risk signals capable of identifying emerging threats and diagnostic failures before adverse events fully materialise (17, 27, 31).

Despite these diverse conceptualisations, no unifying architecture integrates these perspectives or explains how complaints operate within the sociotechnical complexity of hospitals (28, 32, 78). Recent efforts draw on imported socio-technical or behavioural frameworks to develop interventions based on complaints rather than relying on a complaints-specific theoretical model, thereby underscoring the absence of a cohesive foundation (32, 33). The lack of integration limits the strategic potential of complaints and prevents them from informing governance, improvement cycles, and anticipatory risk management. Scholars have already found that complaint systems remain fragmented and poorly aligned with organisational learning processes (10, 34, 35), and that their governance role is recognised more in principle than in practice (36). Despite all these attempts to emphasise complaints as valuable inputs for oversight and governance, integration remains inconsistent and underdeveloped (37), and global safety initiatives continue to call for proactive use of complaints within risk management systems precisely because such utilisation is not yet routine (31).

To address this gap, namely, the absence of an integrative framework to evaluate patients’ complaints in hospitals, this review employs a multidimensional conceptual framework that synthesizes eight interrelated domains of patient complaint management: (1) system consistency in learning from complaints, (2) standard classification of complaints, (3) leadership commitment for organisational learning, (4) effective individual and interpersonal behaviour for organisational learning, (5) organisational change driven by organisational learning, (6) data analytics for organisational learning, (7) proactive risk detection, (8) ethical and legal considerations. Rather than treating these elements as discrete components, the framework positions them as interconnected dimensions that shape how complaints are produced, interpreted, and acted upon within hospital ecosystems.

The main contributions of the paper are summarized in Table 1.

**Table 1:** The main contributions of the paper.

Contribution of the Paper	Description of Contribution	Why It Matters
First integrative eight-domain framework	Synthesizes and provides a unified structure in a field characterized by fragmented scholarship.	Enables hospitals to evaluate complaint systems holistically.
Identification of theoretical and measurement gaps	Emphasizes the absence of validated, multidimensional instruments for measuring and managing complaints.	Guides the development of comprehensive survey tools for empirical research.

### 1.1 Methodological approach

This review adopted a framework-informed integrative approach to synthesize scholarship on patient complaint management in hospitals. Unlike systematic or scoping reviews, which rely on predefined analytical structures and rigid screening protocols, and unlike narrative reviews that often lack a coherent conceptual organising spine (38), integrative reviews allow theoretical patterns to emerge inductively before guiding deductive synthesis. This approach accommodates methodological diversity and supports theory development in conceptually fragmented fields (39–41) and is well-suited to health services research, where constructs are socially produced and contextually embedded (42, 79).

### 1.2 Framework development and review logic

The conceptual framework was developed through iterative inductive–deductive cycles (43–45). Accordingly, the literature search proceeded iteratively rather than linearly, consistent with interpretive review guidance emphasising conceptual refinement over exhaustive enumeration (46, 47).

### 1.3 Search strategy

Searches were conducted across Google Scholar, PubMed, Scopus, and Web of Science. To capture both foundational and contemporary scholarship, the search timeframe was intentionally broad, spanning seminal contributions from the late 1980s through recent empirical and policy-oriented studies. Search terms related to patient complaints, complaint management, organisational learning, organisational culture and change, patient experience, risk detection, and governance were refined iteratively and supported by forward and backward citation tracking.

### 1.4 Literature selection and inclusion criteria

In line with the objectives of an integrative, theory-building review, the literature was selected on the basis of conceptual relevance rather than methodological uniformity or exhaustive coverage. The review prioritised peer-reviewed journal articles and policy reports that examined patient complaints in hospital settings as organisational, governance, safety, behavioural, ethical, or analytical constructs, rather than as isolated service encounters.

Specifically, we sought works that:

- Examined patient complaints in hospital settings;
- Conceptualised complaints as system-level phenomena relevant to organisational learning, governance, patient safety, or ethics;
- Offered theoretical, empirical, or policy-oriented insights with implications for system-level learning or proactive risk detection.

Literature centred primarily on narrow procedural handling, isolated service encounters, or non-hospital contexts, without implications for organisational learning, fell outside the conceptual scope of the review. Although the review did not aim for exhaustive coverage, it synthesised a substantial multidisciplinary body of scholarship. Persistent conceptual fragmentation within this corpus motivated the development of the integrative framework (48–50).

The rest of the paper is organized as follows. The next section expands on the dimensions mentioned earlier and introduces the primary questions for evaluating a complaint management system for each dimension. The final section discusses the findings, including managerial implications and suggestions for future research.

## 2 Integrative multidimensional framework for patient complaint management systems

### 2.1 System consistency in learning from complaints

A key concern in evaluating complaint management systems is whether hospitals receive and resolve complaints in a manner that is consistent with established organisational policies and learning frameworks, systematically applied, and capable of generating strategic intelligence and system-level feedback loops for organisational improvement. Evidence shows that complaint handling remains heavily shaped by organisational structures and procedural design. Early empirical work highlights substantial variation in how hospitals receive, investigate, and resolve complaints, with many systems lacking both procedural consistency and mechanisms for meaningful feedback integration (51). Further studies reveal that complaint processes often operate reactively, relying on fragmented workflows rather than functioning as coherent organisational systems capable of supporting patient-centred learning (5, 6, 10). In response to these shortcomings, recent policy developments have sought to embed complaint management within broader response frameworks. The Patient Safety Incident Response Framework requires that complaints feed into organisational learning, incident analyses, and continuous improvement cycles, thereby formalising a structured process architecture and reinforcing system-level feedback mechanisms (52).

**The central question for this dimension is:** Does the hospital receive and resolve complaints in a manner consistent with established organisational policies and learning frameworks, thereby generating strategic intelligence and system-level feedback loops to support coherent organisational improvement? Or, conversely, is complaint handling primarily reactive and treated as a bureaucratic obligation?

### 2.2 Standard classification of complaints

Standard classification is essential for transforming complaints into meaningful organisational intelligence. Instead of treating complaints as isolated anecdotes, hospitals require a standardised coding system that can detect patterns and safety-critical signals. The development of the Healthcare Complaints Analysis Tool (HCAT) established a foundational taxonomy for structuring complaint content and identifying key risk indicators (17). This taxonomic approach has been extended to digital platforms, where analyses of online hospital complaints draw on classification frameworks grounded in patient-experience principles (53). Evidence from hospital medicine further demonstrates that standardised categorisation enhances the identification of recurrent issues and improves the detection of systemic problems (54). In public hospital settings, integrating taxonomies with structured problem-solving methods provides a consistent and coherent approach to organisational learning, ensuring that coded complaints directly support improvement processes (55).

**The primary questions are:** Does the hospital employ a standardised system for coding complaint content to identify patterns and safety-critical signals, rather than treating complaints as isolated anecdotes? If so, is the coding approach aligned with the analytical and problem-solving methods used to support organisational learning?

### 2.3 Leadership commitment for organisational learning

Effective systems require leadership commitment and a mindset that views complaints as opportunities for improvement rather than reputational threats (56, 76). Managers should actively encourage treating complaints as strategic organisational assets (57–60). Nonetheless, managerial barriers frequently impede their meaningful use, limiting their potential to drive organisational change (35). Managers should adopt a mindset that views complaints as resources for organisational learning, governance improvement, and service quality enhancement, rather than solely as procedural obligations (61, 82, 90).

**The central question for this dimension is:** Does the organisation's culture and leadership demonstrate a commitment to triggering learning and improvement from complaints, or are complaints perceived primarily as a threat?

### 2.4 Effective individual and interpersonal behaviour for organisational learning

Individual and interpersonal behaviours play a critical role in determining whether complaints lead to learning and improvement. Reactions such as defensiveness, emotional responses, or concerns about consequences, substantially shape how staff engage with complaints and whether learning emerges from them (5, 6). Studies further show that interpersonal dynamics involving apology, blame, and acknowledgement strongly influence whether complaints escalate or are resolved constructively (5, 6). Individual and interpersonal behaviours should enhance clinicians’ ability to respond to complaints in ways that support organisational learning (62).

**The central question for this dimension is:** Do individual and interpersonal behaviours contribute to learning and improvement?

### 2.5 Organisational change driven by organisational learning

Complaints become strategically valuable when they catalyse organisational change rather than conclude with the resolution of individual grievances. Evidence shows that complaint analysis should inform corrective actions, loops, and service redesign, ensuring that insights translate into institutional learning (10). At the system level, effective complaint mechanisms contribute to quality improvements across hospital processes by enabling feedback to drive broader organisational change (51, 92). Contemporary empirical studies further demonstrate that structured complaint systems enhance patient satisfaction and perceived service quality, highlighting the link between complaint utilisation and meaningful service improvement (36, 63).

**The central question for this dimension is:** Do complaints catalyse organisational change, or do they end with the resolution of individual grievances?

### 2.6 Data analytics for organisational learning

Digital health innovations increasingly enable complaints to function as assets with predictive and analytical value (80). Statistical models draw on complaint datasets to anticipate the outcomes of quality-improvement initiatives (26). Artificial intelligence–driven analytics enable automated classification and pattern detection, thereby transforming complaints from isolated narratives into scalable organisational intelligence (64, 65, 83, 89). It is essential to integrate complaints into multi-source intelligence systems to support real-time learning, proactive risk management, and system optimisation (66).

**The central question for this dimension is:** Is there a data analytics capability that transforms complaints into data assets with predictive and analytical value?

### 2.7 Proactive risk detection

Complaints are increasingly recognised not merely as expressions of dissatisfaction but as predictive safety signals that reveal emerging risks. Empirical studies show that complaints can precede patient-safety incidents, signalling problems before they fully materialise (67, 77). Contemporary research confirms that routine complaints often contain latent indicators of patient harm and systemic failure (68). Integrating complaints with other safety-intelligence sources, such as incident reports and litigation data, is now widely recommended to support proactive risk detection (66). Evidence from emergency care demonstrates that combining complaints, compensation, and incident records can identify safety “hot spots” and organisational “blind spots,” where risks accumulate unnoticed (69). The literature further argues that complaints expose cultural dynamics, such as blame, defensiveness, and idealisation, that obscure risks from insiders (70). In the safety sciences, complaints are conceptualised as weak signals whose early recognition enables anticipatory action (16), consistent with broader literature showing that systematically captured weak signals can warn of system failure long before major events occur (71). Taken together, this evidence positions complaints as a critical input for organisational foresight and predictive safety intelligence.

**The central question for this dimension is:** Are complaints used as predictive safety signals that reveal emerging risks?

### 2.8 Ethical and legal considerations

Ethical principles are fundamental to complaint handling, which operates at the intersection of patient rights, organisational responsibility, and medico-legal accountability. Complaints serve not only as tools for quality improvement but also as critical mechanisms that demand transparent communication, fair processes, and accessible avenues for redress (61). Complaint management systems must uphold these ethical imperatives by prioritizing openness and procedural justice (7). However, research indicates that compliance with ethical standards and legal requirements is sometimes inconsistent, with moral and procedural obligations falling short of what organisations implement in practice (72).

**The central question for this dimension is:** Are the ethical imperatives of transparency, fairness, and access to redress upheld in complaint handling?

## 3 Discussion

This mini-review presents a framework for repositioning patient complaints as an organisational asset for improvement. By synthesising eight interdependent dimensions, system consistency in learning from complaints, standard classification of complaints, leadership commitment for organisational learning, effective individual and interpersonal behaviour for organisational learning, organisational change driven by organisational learning, data analytics for organisational learning, proactive risk detection, and ethical and legal considerations, the framework emphasizes that complaint management is not a linear administrative task but a system. By conceptualising patient complaints as system-level intelligence, this framework has implications not only for hospital management but also for public health governance, regulatory oversight, and population-level patient safety (84, 85). Previous literature offered valuable but fragmented insights across these domains; this review advances the field by articulating their reciprocal and cyclical relationships. From a systems perspective, system consistency in learning from complaints and standard classification of complaints mutually reinforce one another, enabling proactive risk detection (75). Risk detection both shapes and is shaped by leadership commitment to organisational learning, which in turn co-evolves with effective individual and interpersonal behaviours for organisational learning. These behaviours both drive and are recalibrated by organisational change informed by organisational learning, supported through data analytics for organisational learning. The use of analytics is reciprocally constrained and enabled by ethical and legal considerations, which sustain trust and engagement in complaint systems, thereby reinforcing system consistency and closing the learning loop (81). In the absence of a conceptual model that captures these dynamics, complaints remain siloed and underleveraged; this mini-review provides a foundational basis for an integrative, multidimensional framework for patient complaint management systems.

This mini-review underlines several conceptual shifts required in the field: from treating complaints as administrative transactions to recognising them as knowledge-generating and predictive signals; from fragmented analyses to integrated architectures linking quality, risk, data, ethics, and behaviour; from retrospective review to anticipatory early-warning functions; from technical procedures to systems that embed organisational dynamics; and from individual grievances to systemic voices capable of shaping governance, safety, and accountability (28, 32).

While the proposed framework is intentionally evaluative rather than operational, it is designed to support practical translation through structured assessment and reflection. Each conceptual dimension can be used as an analytical lens for auditing existing complaint management systems, informing governance discussions, and guiding managerial inquiry. In this sense, the framework does not prescribe specific interventions but provides a structured basis for aligning complaint data with organisational learning, quality improvement, and safety governance processes.

This mini-review is intended as a brief conceptual and theory-building contribution to the health services, patient safety, and healthcare management literatures, rather than an empirical or intervention study. Accordingly, the proposed framework integrates existing perspectives but does not specify prescriptive implementation steps. The eight dimensions are evaluative rather than directly actionable, which may limit immediate operational deployment. Future research should therefore translate the framework into applied tools by developing illustrative indicators, audit questions, and metrics, and by linking these to existing instruments such as the Healthcare Complaints Analysis Tool (HCAT) and the Patient Safety Incident Response Framework (PSIRF) (13, 17), and analytics dashboards.

To provide an initial sense of how such translation might be approached, Table 2 presents illustrative, non-prescriptive examples of audit questions, organisational indicators, and governance mechanisms aligned with each framework dimension, intended to support reflection and assessment by health system decision-makers and managers.

**Table 2:** Illustrative audit, organisational, and governance indicators for evaluating patient complaint management systems.

Framework Dimension	Example Evaluative Indicators (Illustrative)
System Consistency in Learning from Complaints	Existence of formal policies linking complaints to organisational learning; regular feedback loops from complaint analysis to quality-improvement committees; documented integration of complaints into learning cycles.
Standard classification of complaints	Use of a standardised taxonomy (e.g., HCAT); proportion of complaints consistently coded; ability to aggregate complaints across departments to identify recurring patterns.
Leadership commitment for organisational learning	Visible leadership engagement with complaint data; inclusion of complaint learning in governance meetings; allocation of resources to complaint-driven improvement initiatives.
Effective individual and interpersonal behaviour for organisational learning	Staff training in constructive complaint responses; evidence of apology, acknowledgement, and reflection in complaint handling; mechanisms to reduce defensiveness and blame.
Organisational change driven by organisational learning	Number of service or process changes explicitly linked to complaint insights; monitoring of post-change outcomes; documentation of learning actions beyond individual case resolution.
Data analytics for organisational learning	Use of dashboards or analytics tools to analyse complaint trends; integration of complaint data with other quality and safety datasets; use of predictive or trend analysis techniques.
Proactive risk detection	Evidence that complaints are used to identify emerging risks; triangulation of complaint data with incident reports or claims; early interventions triggered by complaint patterns.
Ethical and legal considerations	Transparency of complaint procedures; timeliness and fairness of responses; mechanisms ensuring patient access, confidentiality, and procedural justice.

An additional cross-cutting consideration concerns the role of stakeholders and the power dynamics that shape how complaints are interpreted, prioritised, and acted upon within healthcare organisations. Complaints are embedded in asymmetric relationships among patients, clinicians, managers, and regulators, in which authority, professional status, and organisational interests influence which voices are heard and how concerns are framed. Power imbalances may hinder organisational learning when complaints are defensively neutralised, reframed as individual dissatisfaction, or managed primarily to protect reputational or legal interests. Conversely, organisational cultures and governance arrangements that redistribute voice, support psychological safety, and legitimise patient and family perspectives can facilitate learning by enabling complaints to function as credible signals of systemic risk (86). These dynamics do not constitute a separate dimension of complaint management but operate across all eight domains of the framework, shaping leadership responses, individual behaviours, data interpretation, and the translation of complaint insights into organisational change.

The generalisability of the proposed framework should be interpreted with appropriate caution. While the eight dimensions are intended to capture core system-level functions of patient complaint management, their manifestation and relative salience are likely to vary across health systems with different institutional structures, resource levels, and governance arrangements. In highly centralised or well-resourced systems, formal analytics, standardised taxonomies, and regulatory oversight may play a more prominent role, whereas in resource-constrained or decentralised contexts, interpersonal behaviours, leadership commitment, and ethical safeguards may be more critical enablers of learning from complaints. Accordingly, the framework is not proposed as a universal operational model but as a flexible analytical lens that can be adapted to local institutional conditions. Its conceptual transferability lies in supporting context-sensitive interpretation of how complaints function as organisational intelligence within diverse health system configurations, rather than in prescribing uniform implementation strategies.

It should also be noted that the dynamics outlined above are not exhaustive, and additional mechanisms may influence patient complaint management systems. While this mini-review explicitly highlights stakeholder roles and power dynamics as cross-cutting influences on organisational learning from complaints, it does not develop a formal analysis of stakeholder incentives or governance arrangements. Performance-based contracting offers a promising avenue to address this gap, as complaint systems entail information asymmetries and misaligned incentives among patients, clinicians, and managers. While performance-based contracting is well established in supply chain research (73) and increasingly applied in healthcare management (74), its relevance to patient complaint management remains largely unexplored. Future research should therefore examine how incentive-aligned contractual arrangements could strengthen responsiveness, organisational learning, and system improvement in ways consistent with the multidimensional framework proposed in this mini-review.
